# Supplementary material for: Assessing Computational Strategies for the Evaluation of Antibody Binding Affinities
Source: J Chem Theory Comput. 2025 Oct 23;21(21):11271–81. doi: 10.1021/acs.jctc.5c01231 (PMC12613313; doi:10.1021/acs.jctc.5c01231)
Supplement: Supplementary file 1 [file ct5c01231_si_001.pdf]

Supporting information file for the manuscript:

Assessing computational strategies for the evaluation of antibody binding affinities.

Ida Autiero† [1], Damiano Buratto† [2], Fengyi Guo† [3], Wanding Wang [3], Malay Ranjan Biswal [1], Kevin C. Chan [3], Ruhong Zhou [2], Francesco Zonta* [3].

† Equally contributed

* Corresponding author: *francesco.zonta@xjtlu.edu.cn*

1. Department of Biomedical Sciences, Institute of Biostructures and Bioimaging, National Research Council (CNR), 80145 Napoli, Italy.
2. Institute of Quantitative Biology, College of Life Sciences, Zhejiang University, 310058 Hangzhou, China
3. Department of Biosciences and Bioinformatics, School of Science, Xi'an Jiaotong-Liverpool University, Suzhou, 215123, China

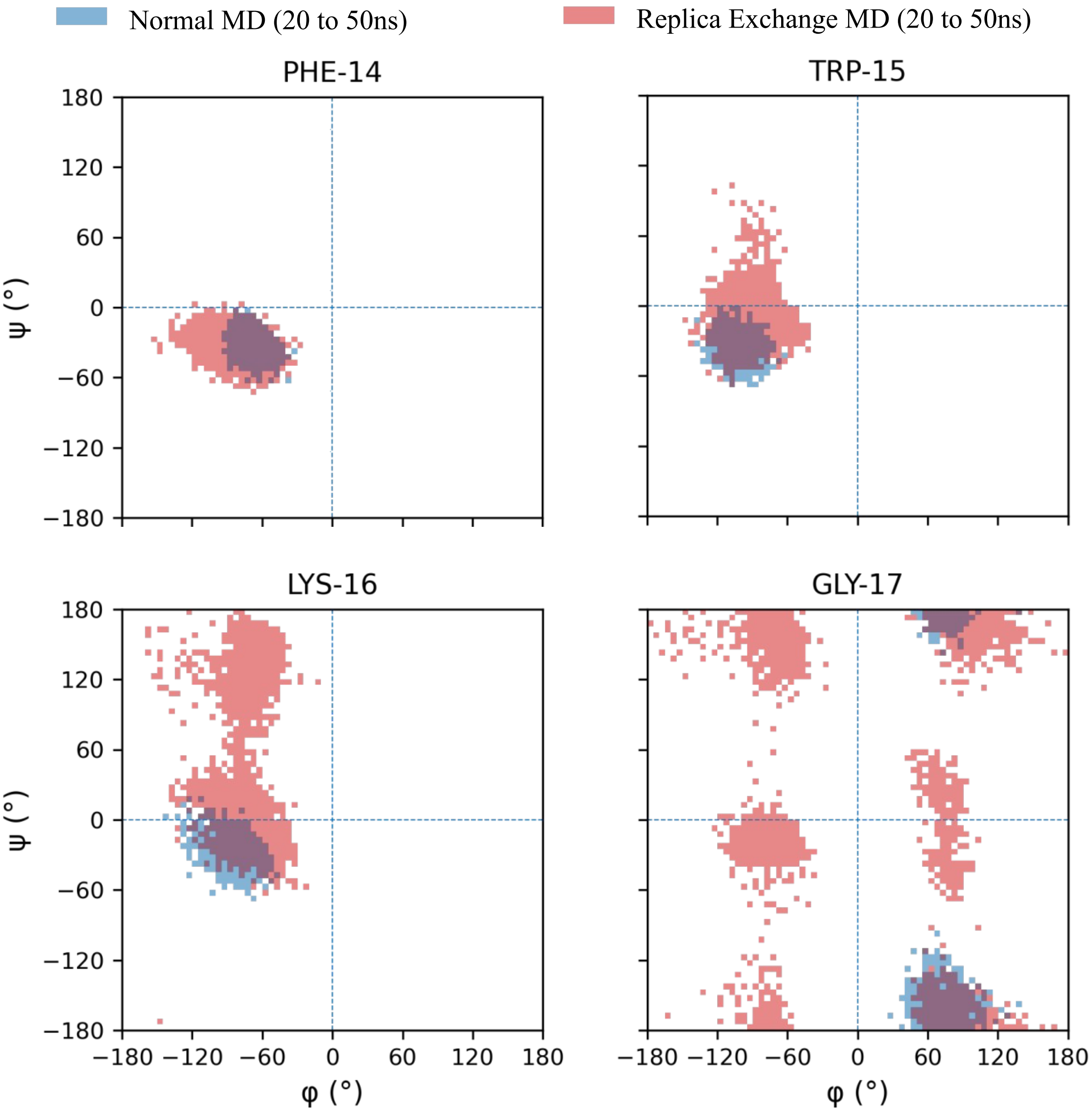

Supplementary Figure 1. Ramachandran plots for normal MD simulations vs T-REMD simulations. The four graphs report the values of the Ramachandran angles for four critical peptide residues (PHE14, TRP15, LYS16 and GLY17). Blue points represent the values for standard MD simulations, while red points for T-REMD. It is evident how in the second case the peptide can explore a vaster configuration space.

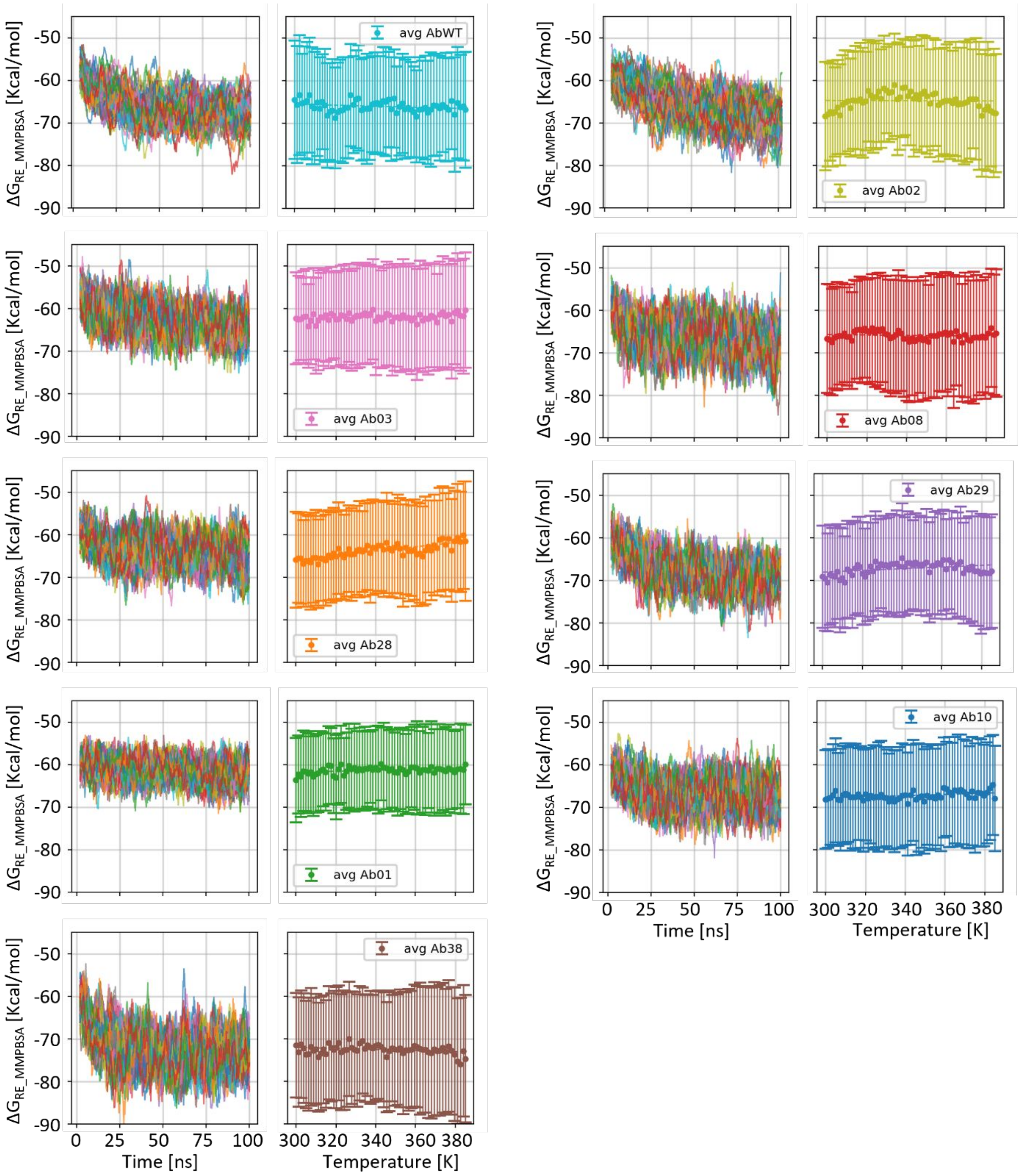

Supplementary Figure 2. Temperature dependence of ΔG in the T-REMD simulations. For each antibody, we report the ΔG values as a function of time for all the different replicas (panels on the left of each subfigure) and the average and standard deviation of the binding affinities calculated in the time period 20-50 ns for all the temperatures considered (panels on the right of each subfigure). Different antibodies thermalize at different times, but generally a plateau is reached after 20 ns. It can also be noticed that temperature dependence is very weak or absent in most cases.
